# Multisensory Information Facilitates Reaction Speed by Enlarging Activity Difference between Superior Colliculus Hemispheres in Rats

**DOI:** 10.1371/journal.pone.0025283

**Published:** 2011-09-26

**Authors:** Junya Hirokawa, Osamu Sadakane, Shuzo Sakata, Miquel Bosch, Yoshio Sakurai, Tetsuo Yamamori

**Affiliations:** 1 Division of Brain Biology, National Institute for Basic Biology, Okazaki, Japan; 2 Cold Spring Harbor Laboratory, Cold Spring Harbor, New York, United States of America; 3 Center for Molecular and Behavioral Neuroscience, Rutgers, The State University of New Jersey, Newark, New Jersey, United States of America; 4 Strathclyde Institute of Pharmacy and Biomedical Sciences, University of Strathclyde, Glasgow, United Kingdom; 5 The Picower Institute for Learning and Memory, RIKEN-MIT Neuroscience Research Center, Department of Brain and Cognitive Sciences, Massachusetts Institute of Technology, Cambridge, Massachusetts, United States of America; 6 Department of Psychology, Kyoto University, Kyoto, Japan; 7 Core Research for Evolution Science and Technology, Japan Science and Technology Agency, Kawaguchi, Japan; Neuroscience Campus Amsterdam, VU University, The Netherlands

## Abstract

Animals can make faster behavioral responses to multisensory stimuli than to unisensory stimuli. The superior colliculus (SC), which receives multiple inputs from different sensory modalities, is considered to be involved in the initiation of motor responses. However, the mechanism by which multisensory information facilitates motor responses is not yet understood. Here, we demonstrate that multisensory information modulates competition among SC neurons to elicit faster responses. We conducted multiunit recordings from the SC of rats performing a two-alternative spatial discrimination task using auditory and/or visual stimuli. We found that a large population of SC neurons showed direction-selective activity before the onset of movement in response to the stimuli irrespective of stimulation modality. Trial-by-trial correlation analysis showed that the premovement activity of many SC neurons increased with faster reaction speed for the contraversive movement, whereas the premovement activity of another population of neurons decreased with faster reaction speed for the ipsiversive movement. When visual and auditory stimuli were presented simultaneously, the premovement activity of a population of neurons for the contraversive movement was enhanced, whereas the premovement activity of another population of neurons for the ipsiversive movement was depressed. Unilateral inactivation of SC using muscimol prolonged reaction times of contraversive movements, but it shortened those of ipsiversive movements. These findings suggest that the difference in activity between the SC hemispheres regulates the reaction speed of motor responses, and multisensory information enlarges the activity difference resulting in faster responses.

## Introduction

The reaction time to multisensory stimuli is often shorter than that to unisensory stimuli. Because the facilitation of reaction by multisensory stimuli occurs even when the upcoming stimulus is unpredictable (e.g., [1]), the facilitation could be attributed to changes in sensory-motor processing rather than to changes in the preparatory state of animals. In our study, we focused on how multisensory information modulates the sensory-motor processing in the superior colliculus (SC) to produce shorter reaction times.

The SC plays a pivotal role in generating orientation movements of the eyes, head, and body in response to sensory stimuli [2]. It possesses a topographically organized spatial map of movement direction [3]–[5]. Neurons in different parts of the SC functionally suppress each other via commissural connections and lateral interactions [6]–[9] under the influence from the basal ganglia [10], which results in the generation of orientation movements to a single relevant direction, suppressing irrelevant movements [11]–[14]. Pharmacological inactivation and electrical stimulation experiments also suggest that the competitive interaction in the SC restricts the reaction speed as well as the direction of a movement [15]–[19].

The SC has ascending and descending inputs from different sensory modalities such as vision and audition [20], [21] and the enhancement of SC activity by coincident multisensory information was shown to facilitate the behavioral responses in cats [22]–[24] and monkeys [25], [26]. However, the combination of different types of sensory information not only enhances but also depresses neuronal activities in the SC depending on the stimulus locations relative to the receptive fields of each neuron [27], [28]. Therefore, multisensory information might modulate competitive interaction within the SC so that behavioral response can be enhanced.

To investigate the competitive interaction in the SC, we analyzed the neuronal activity of one hemisphere of rat SC during the locomotion to ipsilateral and contralateral peripheral stimuli. We focused on SC activities immediately prior to movements, which are most likely to be related with the initiation of movements (i.e., reaction times). We used auditory, visual, and combined audiovisual stimuli to determine the effects of multisensory information on the premovement activity. To test the causal role of SC in contributing to reaction speed, we inactivated neurons in one of the SC hemispheres by injecting muscimol and determined the reaction times to ipsilateral and contralateral sensory stimuli. Studies using rodents as a model animal offer advantages over other species to understand the neural circuit basis of multisensory integration.

## Methods

### Behavioral methods

#### Animal treatment

Handling and euthanasia of animals were done in accordance with the Guide for the Care and Use of Laboratory Animals (NIH publication number 86–23, 1985) and the experimental protocols were approved by the Institutional Animal Care and Use Committee of National Institutes of Natural Sciences and the National Institute for Basic Biology (No. 07A100, No. 08A100 and No. 09A176). We minimized the number of animals used and the extent of animal suffering during all experiments. The subjects were 14–16-week-old male Sprague-Dawley rats (n = 16; SLC, Hamamatsu, Japan), weighing 330–422 g at the start of the experiment. The animals were housed individually in plastic cages in a 12-h light/dark cycle, and water was provided ad libitum. They were fed with sufficient laboratory chow after daily training sessions to maintain more than 90% of their initial weights. They were handled for approximately 1–2 min/day following behavioral training sessions. All the rats were habituated to the chamber and trained to perform behavioral tasks (see below) before surgery.

#### Behavioral apparatus

**Figure 1:**
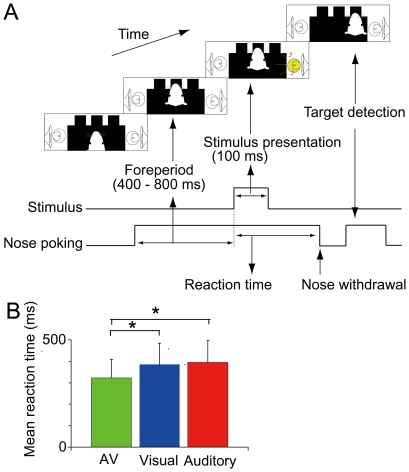
Two-alternative spatial discrimination task based on auditory and/or visual cues. (A) Timing of task events. Nose poking into the central hole initiated a trial. After a variable foreperiod, a cue stimulus was delivered from the left or right, randomly chosen from visual, auditory and audiovisual stimuli. Rats responded to the stimulus by withdrawing from the central hole and selected the direction of the cue stimulus by poking their heads into the hole ipsilateral to the stimulus. (B) Mean reaction time for each type of stimulus across sessions and rats (*p<0.001 in ANOVA and post hoc Tukey test). Error bars, standard deviation.

The apparatus (O'Hara & Co., Tokyo, Japan) was identical to that described previously [1], [29]. Briefly, the main part of the apparatus consisted of three holes for nose poking with infrared photosensors, and a food dispenser in the front wall. Crossing the photosensor by a nose of rat produced TTL pulses which provide the timings of a nosewithdrawing and a nosepoking. Visual and auditory stimuli were presented on the left or right side (Figure 1A). The visual stimulus (1.7 l×) was a white light-emitting diode (LED, 450 mcd, RS components, Japan) covered by a frosted plastic diffuser to generate a homogenous illumination. The auditory stimulus was a broadband white noise (48 db SPL) presented by a loudspeaker placed immediately behind the LED. The intensities were chosen so that similar average reaction times for auditory and visual stimuli can be obtained. All the events were controlled and monitored by a custom-written program (LabVIEW, National Instruments, Austin, TX).

#### Behavioral task

The rats were trained to perform a two-alternative spatial discrimination task, which was slightly modified from that used by Sakata et al. (2004) (Figure 1). Each trial began with nose-poking into the central hole. After a randomized foreperiod from 400 to 800 ms, sensory stimuli were presented on either the left or right side for 100 ms. The sensory stimuli were visual alone, auditory alone, and audiovisual stimuli. The audiovisual stimuli were auditory and visual stimuli that were always simultaneously presented on the same side. The probability that each stimulus was presented was identical. Rats responded by withdrawing their heads from the central hole and poking their noses into the correct hole, which was the same side as that on which the stimulus was presented, within 2.1 s and were immediately rewarded with a food pellet (25 mg). Errors resulted in the turning off of the house light for 3 s (“blackout”) without reward presentation. The same trial was repeated until the animals responded correctly (“correction procedure”). Our correction procedure prevents animals from adopting a behavioral strategy where they always move to a particular direction regardless of stimulus locations and ensures accurate measurement of reaction time to both directions. Reaction time was defined as the duration from stimulus presentation onset to nose withdrawal from the central hole (Figure 1). Although this measure potentially underestimates the initial timing of the responses because the photosensor outputs a digital signal only after the animal's nose is completely undetectable, it reliably detects the multisensory facilitation of reaction time (Figure 1, [1], [29]). Movement time was defined as the duration from nose withdrawal to nose poking into the correct hole. To prevent predictive (premature) responses of the animals, we introduced 16.7% catch trials in all trials of a training and recording session, where no target stimulus was presented and the rats were simply required to maintain nose-poking into the central hole for 1000 ms to receive rewards.

#### Behavioral analysis

All the statistical analyses of behavioral data were conducted using STATISTICA (StatSoft Japan Inc., Tokyo, Japan). For the analysis of data across the conditions with different stimulation modalities, one-way analysis of variance (ANOVA) with repeated measurements and the Tukey test for post hoc comparison were used. All the data are presented as mean ± standard deviation, unless otherwise stated.

### Electrophysiology

#### Fabrication of electrodes and surgery for unit recording

Three animals were used for neurophysiological experiments. Multiunit recording was carried out using a single tetrode composed of four tungsten microwires (20 µm in diameter, California Fine Wire, Grover Beach, CA). To eliminate chewing and movement artifacts, an additional tungsten microwire was also implanted for differential amplification. Fabrication procedures were the same as those previously described [30], [31]. The tip of a microwire bundle was cut with a sharp pair of scissors resulting in about 1 mm of tip protruding from a stainless guide cannula (33 gauge) immediately before surgery. The impedance was approximately in the 500–800 kΩ range (at 1 kHz). A microdrive [31], [32] with the tetrode was chronically implanted under deep pentobarbital-induced anesthesia (50 mg/kg, intraperitoneal injection; Somnopentyl from Kyoritsu Seiyaku, Tokyo, Japan). A rat was placed in a stereotaxic apparatus (Narishige, Tokyo, Japan). The body temperature was maintained by a heating pad. The dorsal surface of the skull was exposed and cleaned. Small stainless screws were fixed in the skull and embedded in dental cement. A small-oval craniotomy was performed on the left parietal bone and the dura was incised. The tetrode was carefully implanted to a depth of 1 mm above the left SC (5.8–6.8 mm caudally from Bregma, 1.4 mm laterally from the midline, and 2.5 mm ventrally from the cortical surface; Paxinos and Watson, 1997). The bottom of the microdrive was fixed on the skull surface with dental cement supported by the skull screws. One screw on the skull over the cerebellum served as the ground. The microdrive was covered with a plastic tube to protect it from scratching by the rats. After surgery, the rats were administered antibiotics and allowed to recover for at least 1 week in their home cages.

#### Neurophysiological recording

Signals were once stabilized by a head amplifier (JH-440J, Nihon Kohden, Japan) and further amplified (×5000) in the main amplifier (AB-610J, Nihon Kohden). After band-pass filtering (500Hz-10 kHz, EW-610J, Nihon Kohden), signals were digitally sampled at 25 kHz with a 12-bit resolution (PXI 6070E, National Instruments). The tetrode was moved daily (approximately 90 µm) using a microdrive after recording sessions, and all the recorded neurons were analyzed.

#### Spike detection and sorting

All the analyses were carried out offline using LabVIEW and MATLAB (Mathworks, Natick, MA, USA). For spike detection, broad-band signals were filtered (0.8–5 kHz). Then, the power (root mean square) of a filtered signal was computed in a 0.2-ms-wide sliding window at 25 kHz [33]. The detection threshold for each channel was defined as more than fivefold the standard deviation of the mean power. When signals from any channels exceeded the threshold, spike waveforms (32 data points) were extracted and stored. For spike sorting, the following parameters were extracted from each spike waveform: energy, peak, valley, and the first two principal components of the waveform. On the basis of these parameters, automated clustering was first performed using KlustaKwik (http://klustakwik.sourceforge.net) [34] and then visually inspected using MClust (http://redishlab.neuroscience.umn.edu/MClust/MClust.html). Clusters were considered as single units only when the following criteria were met: (1) refractory period violations were less than 1% of all spikes and (2) the isolation distance, which was estimated as the distance from the identified cluster to the nearest cluster on the basis of the Mahalanobis distance [35], [36], was more than 20.

#### Spike train analysis

We analyzed single unit data from 43 sessions for each animal during the performance of the spatial discrimination task. Unless otherwise stated, spike trains were smoothed by convolution with a Gaussian kernel (σ = 15 ms) to obtain a spike density function (SDF) for the analysis of the temporal profile of neuronal activity. Prestimulation baseline activity was defined as the mean firing rate during the 100 ms preceding cue onset. Sensory-evoked activity was defined as the mean firing rate during the 100 ms following cue onset. Premovement activity was defined as the mean firing rate during the 100 ms preceding nose withdrawal. To minimize the inclusion of sensory-evoked activity in the calculation of premovement activity magnitude, trials with a reaction time of less than 150 ms, which correspond to 6.2% of the total number of trials, were excluded from the analysis. After exclusion of the data, we were still able to observe the significant facilitation of reaction speed under the audiovisual conditions. Although sensory responses could contaminate the premovement activities in the trials where reaction times were less than 200 ms, our findings were robust even after exclusion of the neurons with visually-evoked activities, which consisted of 12% (21/171) of neurons with movement-related activities. Nevertheless, the mixture of sensory responses and premovement activities in the SC neurons should be a physiologically relevant mechanism to facilitate reaction speed because they could rapidly initiate movements by their greater firing [26]. For the analysis of correlation, the Pearson correlation coefficient *r* and its significance value *p* were calculated.

#### Analysis of sensory responses

A SDF with a smaller kernel (σ = 4 ms) was used for this analysis. Sensory responses were defined as the activities with 2 SDs above the baseline, at a significance level of approximately 5%, for over 15 ms in the periods of 0–100 ms after stimulation onset (first peak) and offset (second peak). These parameters were chosen empirically by closely examining our data set in order to reliably extract any visible sensory-evoked responses. Latency was defined as the earliest time point that crossed this threshold and duration was defined as the period above the threshold. For calculation of the mean firing rates of sensory responses, the periods of 0–40 ms and 50–100 ms after stimulus onset were used for auditory and visual responses, respectively. The same periods were used for calculation of correlation coefficients for sensory responses. A cell was classified as a visual or an auditory cell when it satisfied the above criteria as determined using the data from visual or auditory stimulus conditions alone, respectively. A cell was classified as a multisensory cell when it satisfied the criteria above as determined using the data only from audiovisual stimulus conditions, or when a cell showed both auditory and visual responses.

#### ROC analysis

Owing to the relatively low firing rates of recorded neurons, we used a nonparametric method based on the signal detection theory, receiver operating characteristics (ROC) analysis, to index the modulation of neuronal firing by different task parameters [19], [37]. It calculates the ability of an ideal observer to classify whether a given spike density was recorded under one of two conditions. We indexed the difference between two firing rate distributions by scaling the ROC area between −1 and 1, where 0 reflects no difference between the distributions and the sign denotes whether a neuron fires more under one condition than under the other condition. For time course analysis, we used a 100-ms-wide sliding window with a 20 ms step. Statistical significance (*p*<0.05) was determined with a permutation test of 500 repetitions. For error analysis, false-hit-error (a nose poke into a hole other than the correct hole) trials were analyzed (16.8±8.7 trials with a minimum of four trials). Direction preference index was calculated by ROC analysis between the ipsilateral and contralateral conditions. Premovement activity index was calculated by ROC analysis between prestimulation baseline period and the premovement period defined above.

#### Classification of movement-related neurons

Recorded SC neurons were classified into one of four types on the basis of the sign of premovement activity index and coefficient of Pearson correlation coefficient between reaction time and firing rate in the premovement period. Type 1: Positive premovement activity and positive correlation coefficient. Type 2: Positive premovement activity and negative correlation coefficient. Type 3: Negative premovement activity and positive correlation coefficient. Type 4: Negative premovement activity and negative correlation coefficient. To investigate the characteristics of each neuronal type, we used representative neurons that showed significant premovement activity and correlation coefficient using a threshold of 0.1, removing the data near the boundary of categorization.

#### Multisensory modulation index

To evaluate whether the firing rate under the audiovisual condition was enhanced or depressed compared with that under the unisensory condition, we calculated multisensory modulation index (MSI) in the premovement period for each neuron [25], [38]–[41]. The index was used because pioneering studies indicated that it best describes the multisensory nature of the SC neurons [40]. The index quantified the difference in mean firing rate between the audiovisual stimulus condition and the average of the auditory and visual stimulus conditions using the following formula.
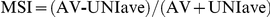
AV is the mean firing rate under the audiovisual condition and UNIave is the average of the mean firing rates under the auditory condition and the visual condition. A positive MSI indicates that a cell increased its activity under the multisensory condition (enhancement), whereas a negative MSI indicates the depression under the multisensory condition.

#### Histology

We used standard histological procedures to confirm all penetrations in the SC. The rats were sacrificed under deep anesthesia (sodium pentobarbital; overdose) and perfused with saline and 4% paraformaldehyde. The brains were removed and 40 µm serial coronal sections were prepared and stained with thionin. The track of a tetrode was identified and the putative boundary between the superficial and intermediate layers in the SC was estimated with the aid of a stereotaxic atlas [42]. The existence of motor-related neurons in superficial layers is consistent with previous studies in rodents [43], [44], though we can not exclude the possibility that our superficial layers were partially overlapped with upper part of intermediate layers due to the uncertainty from the histological reconstruction.

### Pharmacological suppression

Thirteen animals were used for pharmacological experiments. Detailed experimental procedures were described elsewhere [29]. After the animals had mastered the task, cannulae were chronically implanted for muscimol application. Muscimol transiently suppresses the activity of excitatory neurons by activating GABA A receptors on the surface of neurons without affecting passing fibers [15], [45]. The stainless steel guide cannulae (27 gauge, 0.45 mm o.d.) were bilaterally implanted into both left and right SCs using the following coordinates: (i) anterior SC (n = 9), 5.8 mm caudally from Bregma, 1.4 mm laterally from the midline, and 3.2 mm ventrally from the cortical surface, (ii) posterior SC (n = 4), 7.3 mm caudally from Bregma, 1.6 mm laterally from the midline, and 3.0 mm ventrally from the dural surface. Because the results from anterior-biased and posterior-biased suppressions were not quantitatively different, the data from all the animals were pooled for further analysis. The cannulae were fixed with dental cement, and wire stylets were inserted into the guide cannulae to prevent their blockage. The rats were allowed at least one week to recover before starting the experiments. On the test day, the rats were restrained by hand and received microinfusions of either saline or muscimol (25 ng in 0.5 µl of 0.9% saline) through an inner cannula (35 gauge, 0.2 mm o.d.). The rats received simultaneous injections of muscimol into one of SC hemispheres and saline into the opposite side of the SC. Injection of high doses of muscimol (>50 ng) induced an excessive circling behavior toward the side ipsilateral to the site of muscimol injection and prevented the rats from performing the task because they could not poke their noses into the hole contralateral to the site of muscimol injection. Comparable amounts of muscimol were used in other studies in which rat SC was investigated [19], [46]. The tip of the inner cannula extended 0.6 mm below the guide cannula. A 10-µl Hamilton syringe connected to an infusion pump was used to deliver 0.5 µl of muscimol (Sigma) or saline over a period of 2 min using an infusion pump (Model KDS-310, Muromachi Kikai Company, Tokyo, Japan). The inner cannulae were left in place for another 2 min to allow the diffusion of the solution. After the infusion and a 30-min resting period in home cages, behavioral tests were started. Each rat was tested only once per day. Two or three injection sessions were conducted for each rat under the injection condition. The hemisphere for the unilateral muscimol injection was alternately changed among the injection sessions. Bilateral injections of a saline vehicle were carried out in control sessions. After completion of the final test sessions with unilateral muscimol injections, c-Fos immunohistochemistry and thionin staining were conducted to evaluate the effect of muscimol, as described previously [29]. The spread of muscimol around the injection sites was estimated by visually assessing the ranges exhibiting the loss of normal c-Fos staining in the sections.

## Results

### Animal behavior during electrophysiological recordings

Rats were trained to perform a spatial choice task based on auditory and/or visual cue stimuli (Figure 1A, see Methods). To determine the effect of multisensory stimuli on behavior of animals, we first analyzed behavioral data during the electrophysiological recording sessions. The rats performed 186.0±39.1 trials per session with a success rate of 81.1±6.8%. Consistent with our previous reports [1], [29], the mean reaction time to audiovisual stimuli (322.4±86.5 ms) was shorter than that to the visual (386.3±98.7 ms) and auditory stimuli (394.9±101.6) (one-way ANOVA: F_2, 384_ = 22.1, *p*<0.001; with post hoc Tukey test, *p*<0.001, Figure 1B). There were no significant differences in mean reaction time between the visual and auditory stimulus conditions (*p*>0.1), and mean movement time across three stimulus conditions (*p*>0.1).

### Neurons related to locomotion

**Figure 2:**
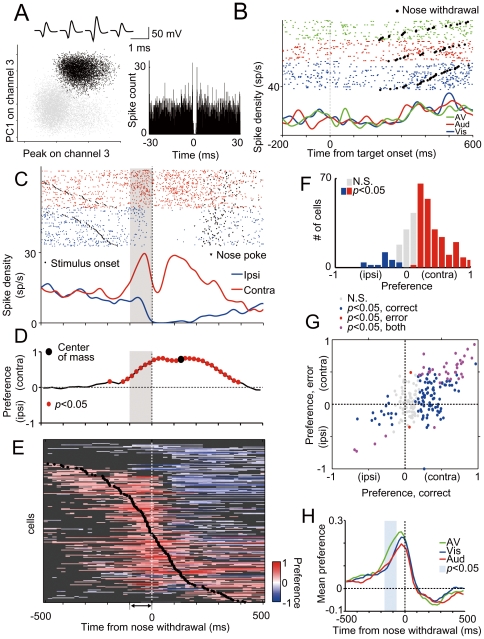
Direction preference preceding locomotion. (A) Example of tetrode isolation for single unit. (Top) Average waveforms of spikes recorded on the four tetrode channels correspond to a cluster in black in the scatter plots below. The scatter plots indicate the peaks of waveforms from channel 3 plotted against principal component 1 (PC1) from channel 3 recorded from a tetrode. (Right) Corresponding autocorrelation functions with a window of ±30 ms. The bin size is 0.1 ms. (B) Rasters and spike density functions (SDFs) aligned to the onset of the target for isolated single unit (black cluster) during spatial discrimination task. The data were derived from only correct trials in responses to contralateral stimuli divided into visual, auditory and audiovisual stimulus conditions. Trials in the raster plot are sorted according to reaction time. (C) The data of the same neuron as above were aligned to movement onset and divided into ipsiversive and contraversive movement trials regardless of the modality of the stimulus. (D) Direction preference index of above unit was calculated using receiver operating characteristics (ROC) analysis for each time point (*p*<0.05, permutation test). (E) Direction preference curves for all cells (225 cells). Each row corresponds to one cell. Cells were sorted by the time of center of mass of significant positive preferences. Red and blue colors indicate preference indices with significant positive and negative values (*p*<0.05, permutation test), respectively, and gray points correspond to insignificant values. (F) Distribution of direction preference index in premovement period across population (225 cells). (G) Preference calculated for correct trials plotted against preference calculated for erroneous trials in premovement period. (H) Direction preferences calculated for each stimulus modality in each neuron and were averaged for each stimulus modality condition. The blue period shows a significant modulation of the preference under the multisensory condition (AV>unimodal, ANOVA with post hoc tukey test, *p*<0.05).

We recorded 225 single neurons (Figure 2A: an example of isolation of a neuron) from the left SC of three rats (71, 76, and 78 cells for each rat) while the rats performed the spatial discrimination task. To examine how SC neurons contribute to the initiation of locomotion in response to sensory stimuli, perievent histograms and rasters were made for each unit, aligned to the stimulus onset for each stimulus. As an example shown in Figure 2B, most of the neurons (73%; 165/225 cells) did not show significant sensory-evoked responses to any stimuli but increased their firing toward the time of initiation of locomotion (nose withdrawal). As clearly seen by aligning the neuronal responses on the onset of locomotion (Figure 2C), the firing rate changed immediately before nose withdrawal from the center hole, suggesting the involvement of SC neurons in the initiation of movement. In addition, the firing patterns were often different between ipsiversive and contraversive movements. This suggests that SC neurons contributed differently to ipsiversive and contraversive movements.

To determine the difference in the activities of individual SC neurons between ipsiversive and contraversive movements, we quantified their differences in firing rate using a direction preference index based on ROC analysis (see Methods). For the example neuron shown in Figure 2C, the direction preference index increased approximately 100 ms before the movement until the completion of the nose poking (Figure 2D). Nearly half of all neurons (111/225 cells, 49.3%) showed significant contralateral preference in the premovement period (0–100 ms prior to nose withdrawal), whereas 6.6% (15/225 cells) showed ipsilateral preference (*p*<0.05, permutation test, Figure 2E, F). The direction preference of the SC neurons starting prior to movement was consistent with that of a previous study of a similar two-choice task in rats using odor stimuli [19]. The time course of the preference index shows that center of the mass of preference curve was in the range from well before to after the initiation of movement (39.55±200.89 ms relative to nose withdrawal) (Figure 2E). This profile was clearly different from that of a previous study using odor cue [19], where the center of mass is only restricted during the locomotion, suggesting the involvement of the SC for sensory-motor transformation for auditory and visual information. Interestingly, 33% of neurons (74/225 cells) showed a distinct ipsilateral preference peak after initiation of the nose withdrawal (shown in bluish color in Figure 2E) as well as an initial peak around the time of initiation of movement. This bimodal preference pattern was not noted in the previous study and suggests that locomotion in our task consists of at least two distinct motor components. Since trajectories of ipsiversive and contraversive movements already differed during the nose withdrawal, these results suggest that the SC activity before nose withdrawal involves in initiation of goal-directed movements. We focused on the first peak in the subsequent analyses to clarify the neural mechanisms underlying the initiation of a locomotion.

To determine whether the contralateral preference in the premovement period corresponds to the stimulus direction or movement direction, we compared the direction preference between correct trials and erroneous trials (Figure 2G). In the correct trials, animals moved toward the direction of the stimulus, whereas animals moved opposite to the direction of the stimulus in the erroneous trials. Thus, if the premovement activity of SC neurons codes the direction of the movement, the preference of direction should be the same between correct and erroneous trials. Indeed, we found a positive correlation between direction preferences for the correct trials and erroneous trials (*r* = 0.62; *p*<0.05), indicating that the direction preference is related to the movement direction rather than the stimulus location. In addition, the time courses of the mean direction preference under different sensory stimulus conditions showed similar patterns (Figure 2H), suggesting that the contralateral preference before the movement is primarily related to the direction of the movement rather than the direction of specific sensory stimuli. Furthermore, the direction preference before a movement was larger under the multisensory condition than under the unisensory conditions (AV>unisensory, *p*<0.05, ANOVA with post hoc Tukey test, Figure 2H), indicating that multisensory stimuli enlarge the difference in the premovement period.

### Relationship between reaction time and premovement activity

**Figure 3:**
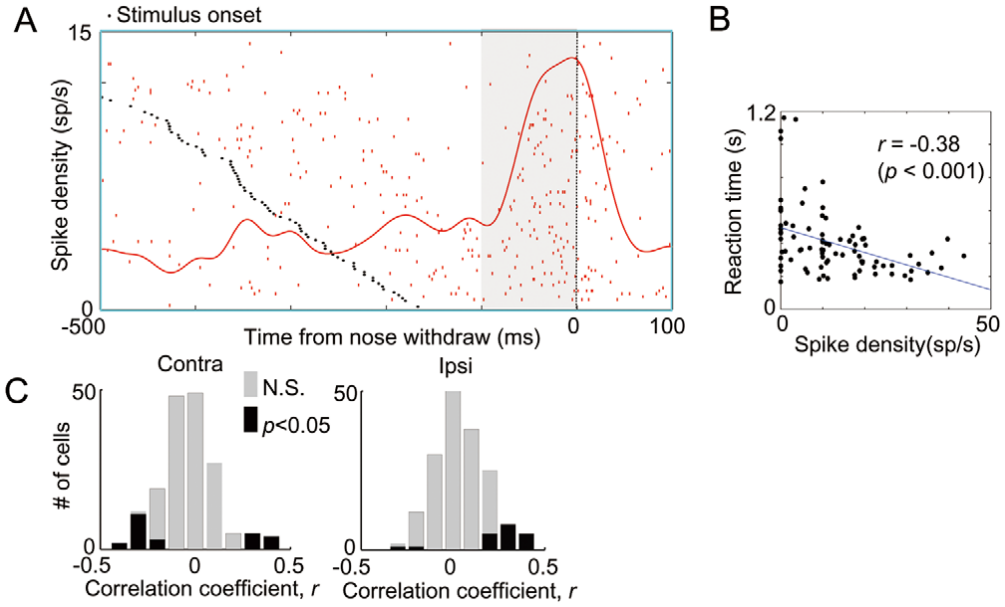
Correlation between reaction speed and firing rates. (A) Raster and spike density function (SDF) for example cell showing negative correlation between behavioral reaction time and firing rate in premovement period. (B) Scatter plots of reaction time and spike density in premovement period of cell. (C) Distribution of correlation coefficients between reaction time and firing rate in the premovement period for contraversive (left) and ipsiversive movements (right).

To investigate the functional role of the premovement activity, we calculated the trial-by-trial correlation coefficient between the firing rates in the premovement period and the reaction times of behavioral responses. We analyzed neurons that showed a significant premovement activity prior to the ipsiversive and/or contraversive movement (171/225, permutation test, *p*<0.05 compared with prestimulus baseline activity). An example neuron in Figure 3A showed higher firing rates in the premovement period when the reaction time was shorter, with a negative correlation coefficient between reaction time and firing rate in the premovement period (*r* = −0.38, *p*<0.001, Figure 3B). As a population, the correlation coefficient between reaction time and firing rate in the premovement period was slightly biased toward negativity (−0.03±0.15, *p*<0.001, *t*-test, Figure 3C left) for the contraversive movement, whereas the correlation coefficient between the reaction time and premovement activity of neurons was positively biased for ipsiversive movements (0.02±0.14, *p*<0.01, *t*-test, Figure 3C right).

**Figure 4:**
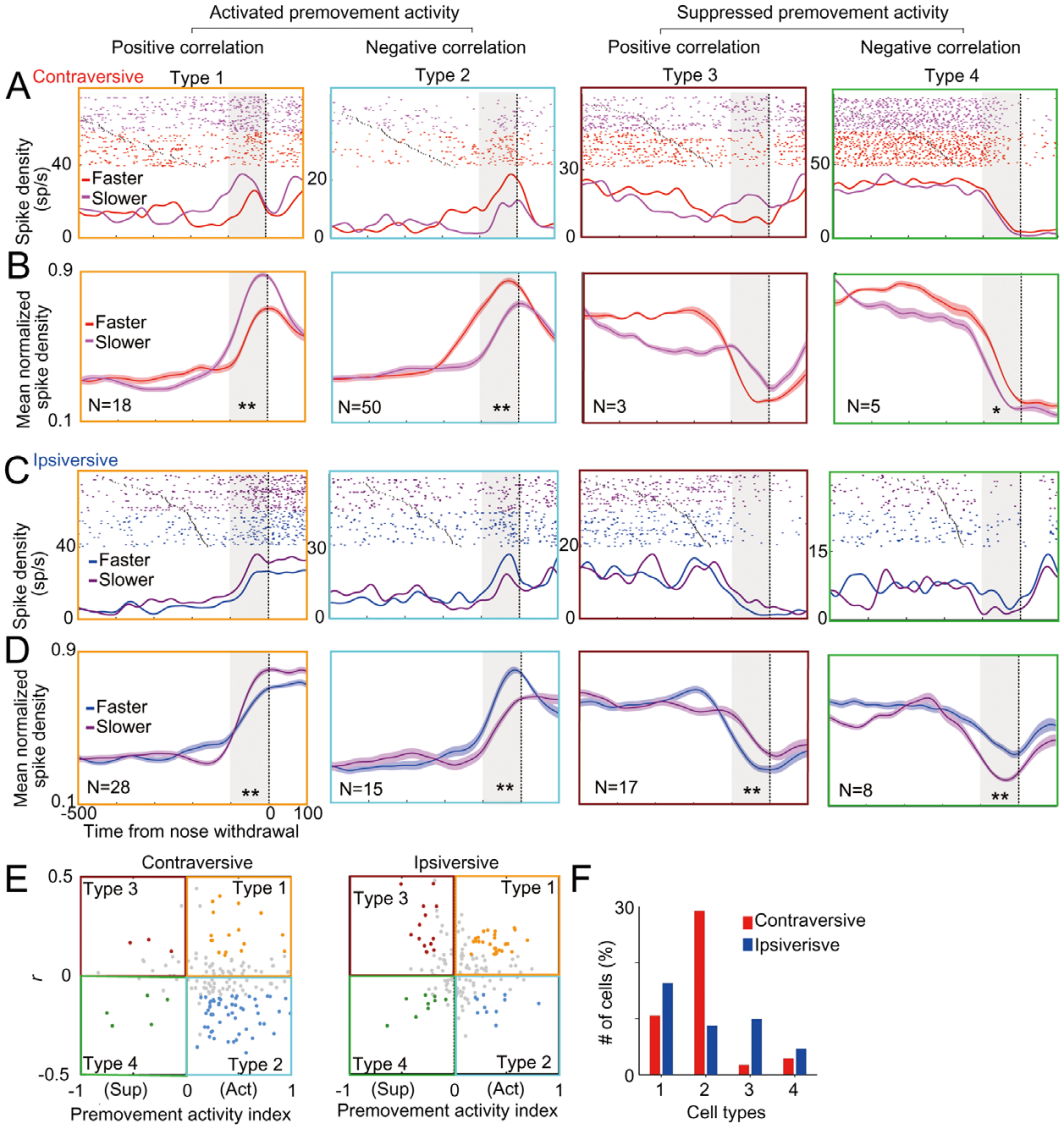
Classification of neurons. Typical examples and their population average of 1–4 types of neuron for contraversive (A–B) and ipsiversive movements (C–D). Trials were sorted according to reaction time and divided into faster half and slower half reaction time trials by the median reaction time and SDFs were averaged for each reaction time group (A, C). The neurons shown in the type 1 and type 2 are the same as those shown in Fig. 2A–C and Fig. 3A, respectively. Population average of mean normalized SDFs of neurons that satisfied criteria (see Methods) for contraversive (B) and ipsiversive movements (D). * *p*<0.05, ** *p*<0.001 in paired *t*-test. (E) Scatter plots indicating the premovement activity index plotted against correlation coefficient between reaction time and firing rate in the premovement period. The premovement activity index is a normalized premovement activity so that 0 equals the spike density during the prestimulus baseline period (see Methods). The four colors of dots are used to code neuron types 1–4 that fall in one of four quadrants in the scatter plots with defined criteria (see Methods). (F) Percentage of each type of neuron for ipsiversive and contraversive movements (n = 171).

We noticed that some neurons increased their firing rates before movement, whereas other neurons decreased their firing rates before movement. To determine whether activation or suppression during the premovement period contributes to positive or negative correlations, we divided the cells into one of four types on the basis of their premovement activity and correlation coefficient (Figure 4). In the categorization, type 1 and 2 neurons are activated before movement, whereas type 3 and 4 neurons are suppressed. In terms of correlation coefficients, type 1 and 3 neurons have positive correlation coefficients, whereas type 2 and 4 have negative ones. In summary, the feature and presumable role of each type of neurons are as follows:

- Type 1 neurons: Activation of the premovement activity brakes behavioral responses.
- Type 2 neurons: Activation of the premovement activity accelerates behavioral responses.
- Type 3 neurons: Suppression of the premovement activity brakes behavioral responses.
- Type 4 neurons: Suppression of the premovement activity accelerates behavioral responses.

Typical examples of each type of neuron for contraversive and ipsiversive movements are shown in Figures 4A and C, respectively. All trials were divided into one-half with a short reaction time and the other half of a long reaction time so that the relationship between reaction time and the time course of SDFs can be seen.

In total, 69.6% of cells (119/171) satisfied our criteria of the classification for either ipsilateral or contralateral movements (see Methods) and the rest of the cells that have insignificant values near boundaries were removed from further analysis (colored in gray in Figure 4E). To characterize the temporal profile of neurons categorized into each type, we averaged the SDFs of neurons in each type (Figures 4B and D) that satisfied the criteria (colored cells in Figure 4E). The mean SDFs showed that the premovement activity changes immediately before movement (∼100 ms) and has systematic relation with reaction speed.

For contraversive movements, about one-third of neurons (50/171) were categorized into type 2 and a smaller number of neurons (18/171) were categorized into type 1 (Figure 4C). Only a few neurons satisfied the criteria for categorization into types 3 and 4 (3/171 and 5/171, respectively). On the other hand, for ipsiversive movements, the number of type 2 cells was much smaller (29.2% to 8.7%; contraversive to ipsiversive movements), whereas the numbers of type 1 and type 3 neurons were larger (type 1, 10.5 to 16.3%; type 3, 1.7 to 9.9%) than those for the contraversive movements (Figure 4F). In the type 1 neuron, a smaller activation in the premovement period resulted in a faster reaction, whereas a greater suppression of the type 3 neuron in the same period resulted in a faster reaction (Figure 4C, D). Thus, in both types of neuron, the decrease in premovement activity should lead a faster reaction for ipsiversive movement. Taken together, our results suggest that greater activity in SC neurons accelerates the initiation of contraversive movements and decelerates the initiation of ipsiversive movements.

To determine whether the classified types of premovement activity are preserved in both the ipsiversive and contraversive movements, we mapped the cell types defined by the contraversive movement against those defined by the ipsiversive movement in scatter plots (Figure S1). The cell types defined by the contraversive movement were not consistent with those defined by the ipsiversive movement. Furthermore, there was no significant correlation between the correlation coefficients for ipsiversive and contraversive movements (*r* = 0.11, *p*>0.1), suggesting that different subsets of neurons contribute to the initiation of movements toward opposite directions.

### Modulation of premovement activity by multisensory information

**Figure 5:**
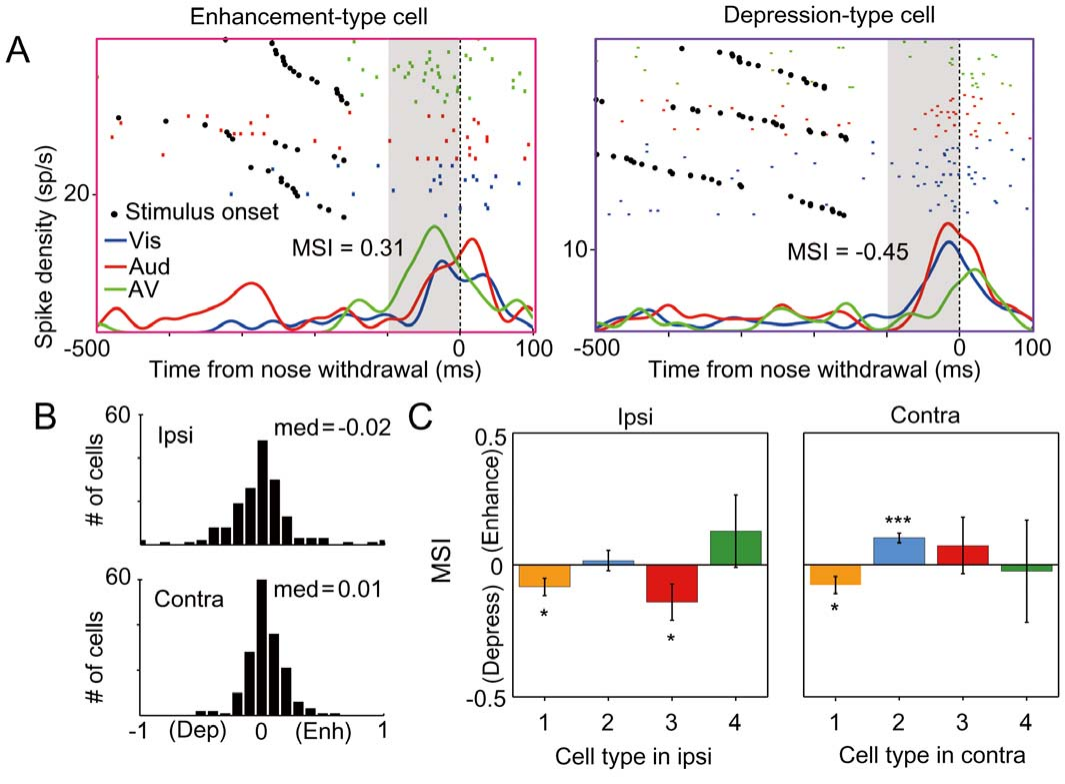
Enhancement and depression in premovement activity by multisensory stimuli. (A) Examples of neurons showing enhanced or depressed premovement activity for contraversive movement under audiovisual (AV) stimulus condition compared with those under visual (Vis) and auditory (Aud) stimulus conditions. MSI (multisensory modulation index, see Methods) in the premovement period for each neuron is shown. (B) Graphs show the distributions of MSIs in the premovement period for the ipsiversive and contraversive movements. (C) Mean MSI divided into cell type groups and directions of the movements, showing significant deviation from zero in some cell types (* *p*<0.05, ** *p*<0.01, *** *p*<0.001 in *t*-test).

We next examined whether the activation or the suppression in the premovement period might be further modulated by multisensory information to produce faster movements. We analyzed SDFs of the data divided into different modality stimulus conditions. As shown in examples in Figure 5A, some neurons showed enhancement or depression of the premovement activity under the audiovisual condition compared with those under the unisensory conditions. To determine the magnitude of the modulation caused by multisensory information, we indexed the modulation in the premovement period by comparing firing rates under the audiovisual condition with mean firing rates under the unisensory conditions (see Methods). In all the populations of neurons (Figure 5B), the distribution of multisensory modulation prior to the contraversive movement was slightly biased toward enhancement (0.03±0.16, *t*-test, *p*<0.05), whereas the index prior to ipsiversive movements was not significantly biased from zero (−0.02±0.26, *t*-test, *p*>0.1). We next examined which types of cell were modulated to show enhanced and depressed activations (Figure 5C). In the case of the contraversive movement, the type 2 cells showed an enhanced activation under multisensory condition (*p*<0.05, *t*-test), whereas type 1 cells showed a suppressed activation under the multisensory condition (*p*<0.05). On the other hand, in the case of the ipsiversive movement, the activities of type 1 and type 3 neurons were mostly suppressed under the audiovisual stimulation condition (*p*<0.05). Thus, multisensory information enhanced the premovement activity of acceleration-type neurons (type 2) and depressed that of suppression-type neurons (type 1 and type 3).

**Figure 6:**
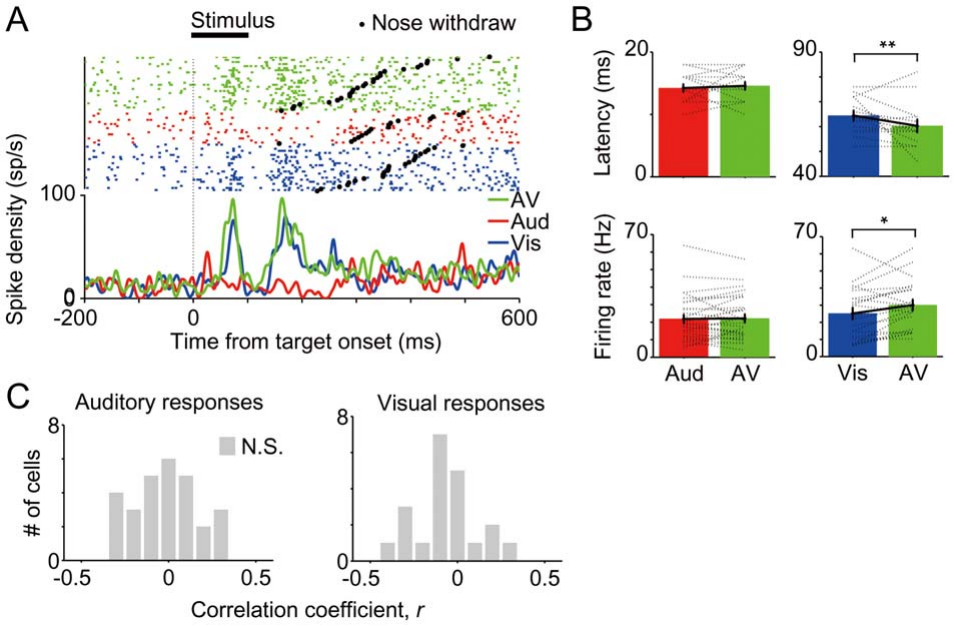
Sensory-evoked responses. (A) Examples of a sensory-motor neuron showing visually-evoked responses. Raster plots and spike density functions (SDFs) were aligned to stimulus presentation. Trials in the raster plot were sorted according to reaction time. The data were derived from only correct trials in response to the stimuli presented to contralateral stimuli. (B) Comparisons of latencies and firing rates of sensory-evoked responses between unisensory and multisensory stimulus conditions in the multisensory cells. The dotted lines show latencies and mean firing rates of evoked responses for each cell (n = 29, 21 for auditory- and visually-evoked responses, respectively). The histograms and error bars show the means and standard error across cells, respectively. *p<0.05, **p<0.01 in paired *t*-test. (C) Distribution of correlation coefficients between reaction time and firing rate in the sensory-evoked response periods of visual or auditory responsible cells (n = 32, 17 cells for auditory and visual responses, respectively).

Among neurons that had any significant premovement activity, a subset of neurons (32.16%; 55/171) also showed significant sensory-evoked peaks in the spike histogram aligned to the onset of stimulus presentation (i.e., sensory-motor neurons, Figure 6A), although one of three rats (# 674) did not show any sensory responses during the task likely due to the electrode location.. Multisensory stimuli enhanced magnitude of the visually-evoked responses (*p*<0.05, paired *t*-test, Figure 6B) and shortened the latency of the visual responses (*p*<0.01, paired *t*-test). However, the MSI in the sensory response period did not correlate with that in the premovement period (*r* = 0.074, *p*>0.1) of the same cell. In addition, reaction time and firing rate in the sensory response period did not show any significant correlations (Figure 6C), suggesting that magnitudes of sensory evoked activities in each cell do not significantly influence on the reaction speed.

Histological verification of electrode position indicated that each type of neuron was recorded from the superficial to deep layers of the SC (Figure S2). Type 1 and type 2 neurons were consistently found in all three animals. Type 3 cells for the ipsiversive movement were mostly found in one of the animals (# 674). The tetrode track of this animal was found in the rostral part of the SC (approximately 5.8 mm from Bregma), which represents visual and auditory information in the nasal space [4]. The result suggests that the rostral SC suppresses the initiation of locomotion, which is consistent with the results obtained in the primate SC [47]–[49]. In addition, since the spatial distribution of each type of neurons was clustered in the SC (Figure S2), different categories could represent distinct functional populations in the SC.

### Effect of SC inactivation on reaction facilitation

**Figure 7:**
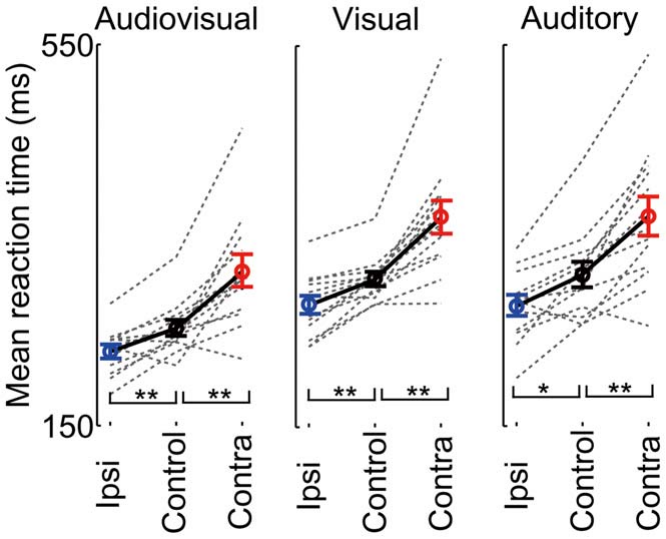
Inverse effects of muscimol on ipsiversive and contraversive movements. Distribution of reaction times for ipsiversive movement and contraversive movement after unilateral muscimol injections. Control is the mean reaction times of the pooled data from left and right movements after bilateral saline injections of each animal. The dotted lines denote mean reaction time for each injection condition of each animal. The circles and error bars denote the means and standard error across animals, respectively. *p<0.01, **p<0.001 in paired *t*-test comparisons using the Bonferroni correction.

**Figure 8:**
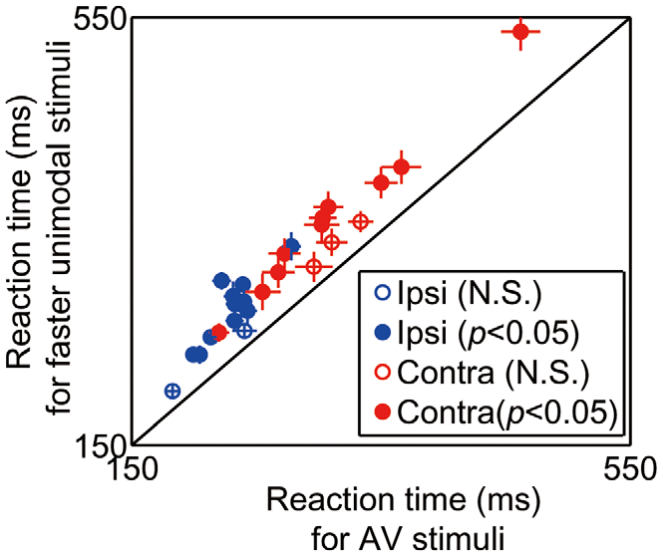
Intact multisensory facilitation after muscimol injections. Mean reaction times for audiovisual stimuli were plotted against mean reaction times for corresponding faster unisensory stimuli for ipsilateral (blue) and contralateral choices. Filled circles indicate individual animals in which reaction times for audiovisual stimuli are shorter than those for unisensory stimuli (*p*<0.05, *t*-test). Error bars, ± standard error.

To determine the relationship between behavioral reaction time and activity of SC neurons, we conducted pharmacological inactivation experiments. We injected muscimol into one SC hemisphere and saline into the other SC hemisphere and examined the performance of the rats in the spatial discrimination task. The inactivated areas were the deep and superficial layers of the anterior half or posterior half of the SC, as verified by c-Fos expression analysis (Figure S3). For the control condition, rats' accuracy was 84.0±4.9%. After muscimol injection, success rate decreased in trials of the contraversive movement (75.6±8.0%, *p*<0.0001, paired *t*-test, with Bonferroni correction) but increased in trials of the ipsiversive movement (86.3±4.2%, *p*<0.001). The reaction times of the animals in which muscimol was injected into the contralateral side were always longer than those under the control condition under any stimulus-modality conditions (*p*<0.001, paired *t*-test, with Bonferroni correction, Figure 7) and the reaction times of animals in which muscimol was injected into the ipsilateral side were shorter than those under the control condition under any stimulus-modality conditions (*p*<0.01). These results are consistent with the idea that the contributions of SC to ipsiversive and contraversive movements are in contrast (i.e., suppressive and accelerative, respectively). We next examined whether the time of reaction to the audiovisual stimuli is still shorter than those to the unisensory stimuli in the ipsilateral and contralateral movements (Figure 8). The reaction times to the audiovisual stimuli are significantly shorter than those to the unisensory stimuli for both movements (10/13, 10/13 individual animals, *p*<0.05, *t*-test, ipsilateral and contralateral, respectively).

## Discussion

We examined the role of SC neurons in behavioral reaction to unisensory and multisensory stimuli. We found that neuronal activity in the SC immediately before a movement positively and negatively correlated to the initiation of the movement to the ipsilateral and contralateral sides. In addition, multisensory stimuli enhanced premovement activity of a certain population of SC neurons for the contraversive movement, whereas multisensory stimuli suppressed premovement activity of another population of neurons for the ipsiversive movement, both of which resulted in a faster behavioral reaction to the stimuli. These findings suggest that the difference in activities among SC populations, which encode target and nontarget directions, controls the initiation of movements toward sensory targets. Previous researches have suggested that the variability of reaction time can be partly explained by the preparatory state of animals, affected by the competitive interaction in the SC and inputs from the basal ganglia [47], [49]–[53]. Our data extend this view by showing that reaction time depending on bottom-up sensory-motor processing is also affected by the difference in activities among SC neurons, and multisensory information enlarges the activity difference, resulting in a faster behavioral reaction.

### Role of rodent SC in spatial discrimination

We showed that SC of rats is responsive to auditory, visual and multisensory stimuli during the spatial discrimination task, which were consistent with the previous studies using anesthetized and freely-moving rodents [39], [54], [55]–[57] and other species [22], [24]–[26], [58]–[62]. Our data also showed that a large population of neurons in the SC have movement-directional preference immediately before locomotion in response to visual and auditory stimuli as similar to saccade task using primates [63], [64]. The existence of direction preference before a movement is consistent with a previous study of rodents performing the direction choice task using olfactory stimuli [19]. In that study, the direction preference of activity appeared more prominently during the movement, whereas the contralateral preference of activity in our study was prominent before a movement (Figure 2E). Because the SC has a spatial map of visual and auditory information [65], sensory-motor transformation could occur in the SC in our task [66], whereas different neural mechanisms might function in the decision of movement direction based on the odor identity (e.g., [67]).

### SC activity and reaction speed

Psychological studies of human reaction time have led to mathematical models in which sensory information is accumulated over time and a decision is made when it reaches the threshold [68]–[72]. Indeed, some physiological studies using primates suggest that the accumulation of sensory evidence in certain brain areas to a threshold triggers a movement [73]–[75].One general class of models, known as accumulator models predicts two outcomes: (1) the firing rate immediately before movement is constant irrespective of whether the reaction time is short or long, and (2) the rate of rise of premovement activity is steeper in short-reaction-time trials than in long-reaction-time trials [73]. Our data clearly deviated from both of these predictions (Figures 4A–D and the rest of our data), suggesting that individual SC neurons did not have a certain threshold activity to trigger the movement in our task. It is likely that the short-duration stimulus (100 ms) we used did not allow the gradual accumulation of sensory information in the SC, because the accumulation process depends on exposure duration to the stimuli [74], [76].

We found that the reaction times correlated with the firing rates of SC neurons in the fixed premovement period. Although the correlation coefficient of each cell was not large, there were significant biases toward negative and positive values in the contraversive and ipsiversive movements (Fig. 4), respectively. The weak correlation was consistent with previous studies of perceptual decision tasks in primates [51], [53], which is likely to be due to the fact that moving directions are coded by a large number rather than a small fraction of neurons in the SC [77], [78].

### Competitive interaction in SC

Our detailed analysis revealed two types of SC neurons in terms of the inhibition of movements. One is type 1 neurons, which inhibited the initiation of a movement by their activation immediately prior to movements. Thus, they could be acting competitively with other acceleration-type neurons (i.e., type 2 neurons) for triggering a movement. The other is type 3 neurons, which could inhibit movements during the prestimulus and premovement periods. This type of neurons is similar with so-called fixation neurons in primates, which fire tonically when a monkey actively fixates on the target spot and pause firing during the execution of saccadic eye movements [11], [48], [64], [79]. Indeed, we observed that type 3 neurons showed pattern of firing clearly complementary to that of type 2 neurons, which suggests that the pause of activity of type 3 neurons allows transient bursting activation of type 2 neurons immediately prior to movements. Inputs from other brain regions such as the basal ganglia may also contribute to the activation and suppression of SC neurons [10], [80]–[84]. These mechanisms could synergistically work to distinguish the specific population of SC neurons that are relevant to directional movements.

As we assumed that the hemispheres of SC functioned symmetrically in our task, we expect that one hemisphere of SC is accelerative for the contraversive movement and the other hemisphere of SC is suppressive for the same movement. Therefore, the activity difference between the two hemispheres of SC might restrict the reaction toward peripheral targets. This assumption is supported by a recent study suggesting that the activity difference among different populations of SC neurons reflects the certainty of direction choice in primates [85].

### Role of SC in multisensory behavioral enhancement

The enhancement of premovement activity by the audiovisual stimuli was evident in the type 2 neurons, which showed increased premovement activity prior to contraversive movements. This finding was consistent with the results of a previous study of the oculomotor system in primates [26]. In addition to the enhancement in premovement activity of a subset of neurons, we found that the premovement activity of other populations of neurons was depressed under the audiovisual stimulus condition (Figure 5C). Although it is known that the multisensory depression of neuronal activity occurs when unisensory information is not temporally or spatially consistent [65], the situation in which we observed the multisensory depression had consistent unisensory information. The depression was evident in the neurons whose activity is supposed to suppress the generation of the movement (i.e., neurons with positive correlation between reaction time and premovement activity, Figure 5C). Thus, we suggest that multisensory depression, as well as enhancement, plays an important role in enlarging the difference in neuronal activity between the SC hemispheres, which is a mechanism to produce a faster behavioral response to audiovisual stimuli. One possible source that can enhance and depress the premovement activity of particular populations in the SC is selective descending inputs from multisensory cortical areas (e.g., [29], [86], [87]).

The SC has been considered as a brain region that is causally essential for integrating visual and auditory information, as demonstrated by a study of excitotoxic lesion of the SC in cats [24]. On the other hand, our present study showed that unilateral SC inactivation did not affect the facilitation of reaction speed to the multisensory stimuli (Fig. 8). Because we have observed strong behavioral defects in responses to any type of contralateral stimulus, it is unlikely that only multisensory neurons were intact in this situation. One possible explanation for the lack of effect on multisensory responses is that the spatial range of inactivation may have not been sufficient to suppress the integrative ability of the SC. Because we targeted the inactivation of the anterior half or posterior half of one hemispherical SC, the intact part of the SC might still have the capability to produce an enhanced behavior. However, this is contradicted by the finding of a previous study that both rostrally and caudally biased lesions of one hemisphere cause a complete loss of multisensory behavioral enhancement [24]. Therefore, it may be more likely that the SC might not be necessary for integrating auditory and visual information for facilitating reaction speed. Burnett et al. have shown that the enhancement of spatial detection accuracy by multisensory integration is eliminated by SC lesions [24]. However, no researchers have examined the effect of SC lesions on the facilitation of reaction speed by multisensory stimuli. It is possible that different neural networks are responsive for accurate sensory detection and rapid response, respectively. Indeed, we previously showed using the same technique with this study that the inactivation of the secondary visual cortex (V2L) suppresses the facilitation of reaction speed [29]. This is a line of evidence that shows that multisensory integration for the facilitation of reaction speed occurs not in the SC but in the V2L.

## Supporting Information

Figure S1 **Relationship of cell types between contraversive and ipsiversive movements.** Cell types defined by contraversive movement were mapped on the scatter plots in Figure 4B (defined by ipsiversive movement).
(PDF)

Figure S2 **Spatial distribution of 4 types of neurons in the superior colliculus (SC).** (A) A Nissl-stained coronal section of an animal (# 673) shows the recording track of a tetrode. Tetrode tracks are indicated by a black line in the right diagram (adapted from Paxinos and Watson, 1986). (B) Depth profile of each type of cell for contraversive movement (n = 83, top) and for ipsiversive movement (n = 72, bottom). Each plot indicates the relative depth of the cell recorded in three animals indicated (# 672–674). Gray horizontal lines indicate the putative boundary between superficial and intermediate layers in the SC. Each color indicates one of four types of neuron defined by contraversive movements. The locations of the recording electrodes from three animals are indicated by blue circles in the dorsal view of the rat SC (bottom left).
(PDF)

Figure S3 **Evaluation of the region in the superior colliculus (SC) suppressed by muscimol.** (A) Representative photomicrographs of the SC of rats that performed the spatial discrimination task after injecting saline (left) and muscimol (right). # indicates the location of injection tip. Black lines cover the regions where c-Fos expression in glial cells was observed. Black dotted lines indicate the border of deep layers of the SC. Scale bars, 1 mm. (B) Coronal diagrams (adapted from Paxinos and Watson, 1986) showing the location of injection sites and the spread (shown by shaded areas) of muscimol within anterior (left) and posterior (right) parts of the SC after anterior-biased (n = 9) and posterior-biased (n = 4) injections, respectively. The injection sites are indicated for the saline-injected (left) and the muscimol-injected (right) hemispheres. Each shaded area indicates the area where suppression of c-Fos expression was observed in each animal.
(PDF)
