# Supplementary figures and images for: Multisensory Information Facilitates Reaction Speed by Enlarging Activity Difference between Superior Colliculus Hemispheres in Rats
Source: PLoS One. 2011 Sep 26;6(9):e25283. doi: 10.1371/journal.pone.0025283 (PMC3180293; doi:10.1371/journal.pone.0025283)

## Supplementary Figure 1

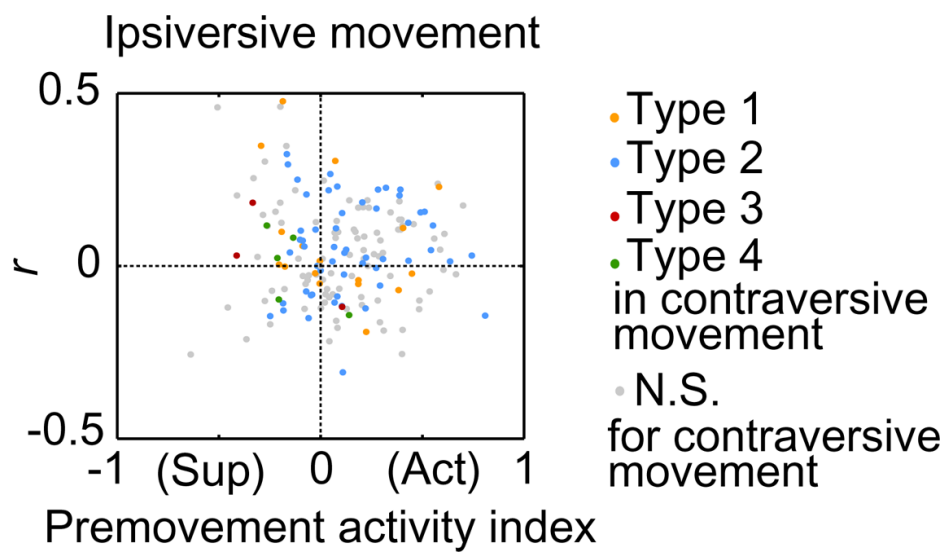

Supplement: Figure S1 — Relationship of cell types between contraversive and ipsiversive movements. Cell types defined by contraversive movement were mapped on the scatter plots in Figure 4B (defined by ipsiversive movement). (PDF) [file pone.0025283.s001.pdf]

Supplementary Figure 2

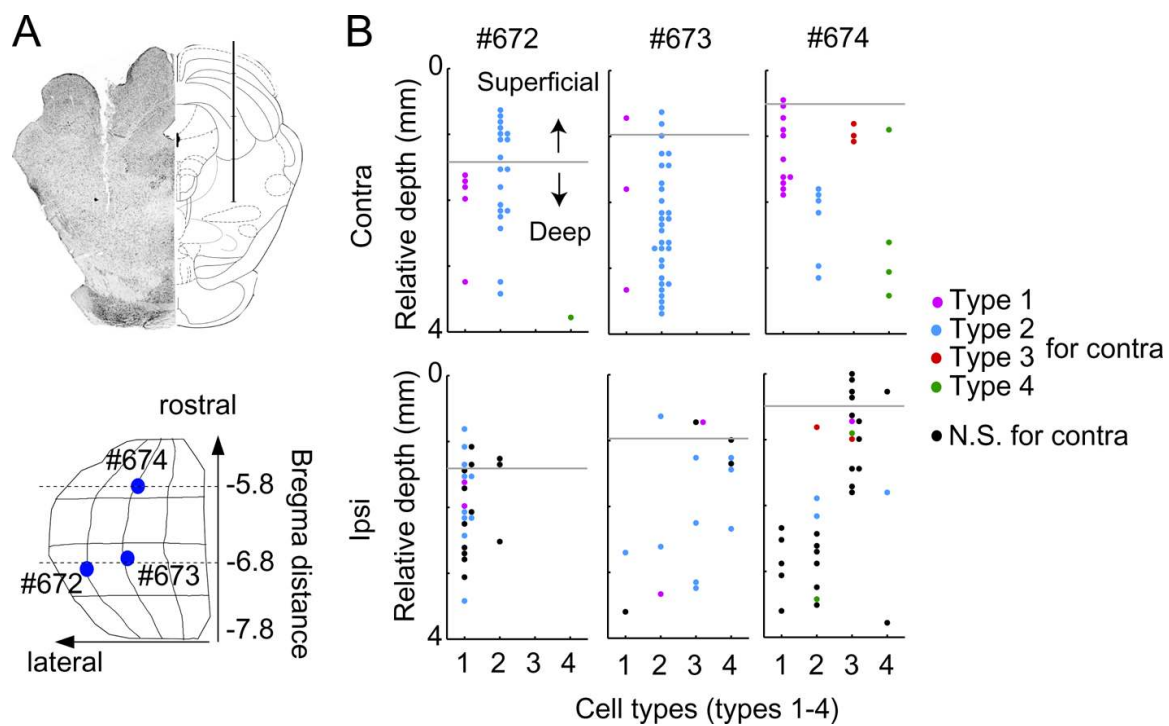

Supplement: Figure S2 — Spatial distribution of 4 types of neurons in the superior colliculus (SC). (A) A Nissl-stained coronal section of an animal (# 673) shows the recording track of a tetrode. Tetrode tracks are indicated by a black line in the right diagram (adapted from Paxinos and Watson, 1986). (B) Depth profile of each type of cell for contraversive movement (n = 83, top) and for ipsiversive movement (n = 72, bottom). Each plot indicates the relative depth of the cell recorded in three animals indicated (# 672–674). Gray horizontal lines indicate the putative boundary between superficial and intermediate layers in the SC. Each color indicates one of four types of neuron defined by contraversive movements. The locations of the recording electrodes from three animals are indicated by blue circles in the dorsal view of the rat SC (bottom left). (PDF) [file pone.0025283.s002.pdf]

Supplementary Figure 3

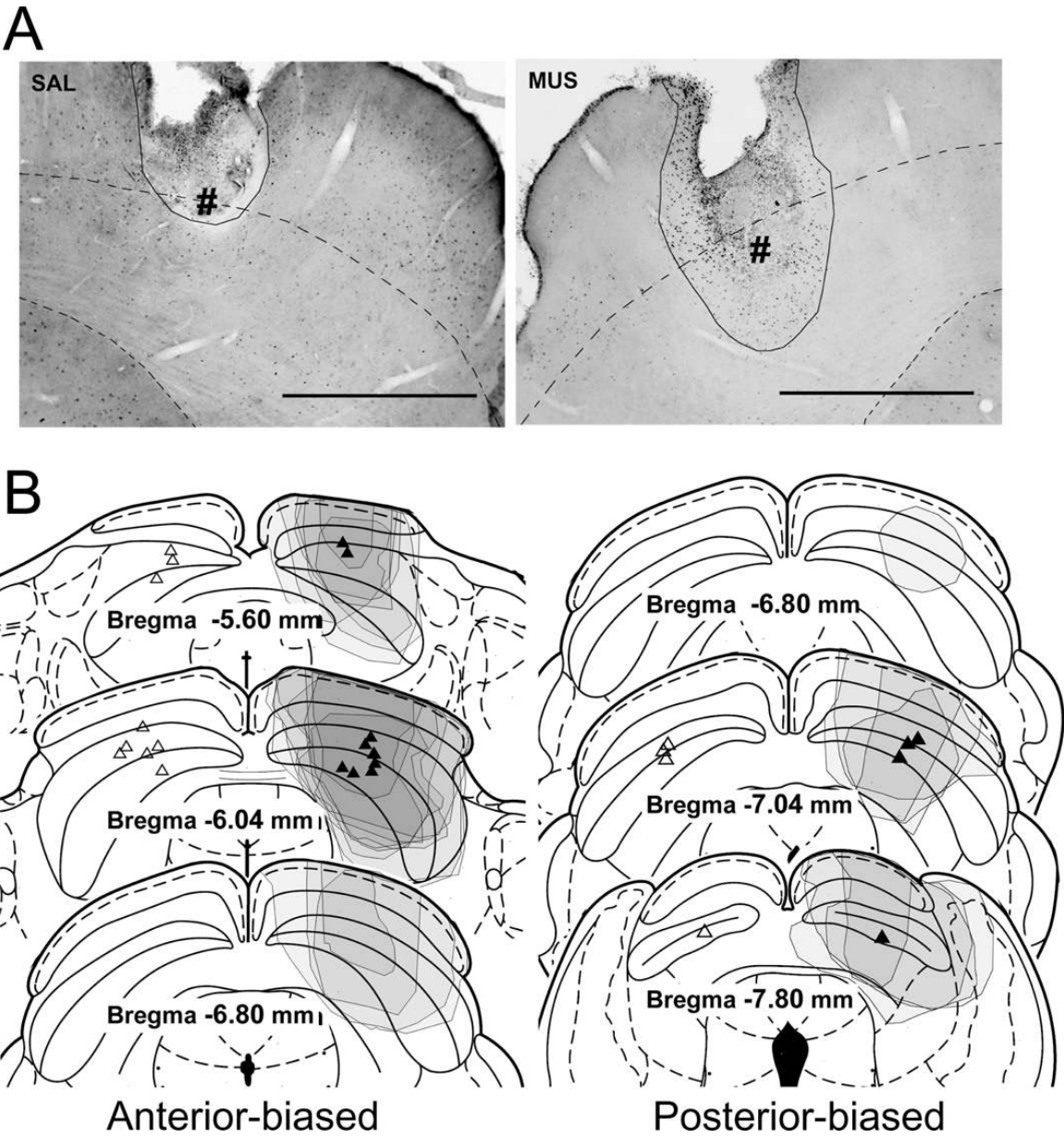

Supplement: Figure S3 — Evaluation of the region in the superior colliculus (SC) suppressed by muscimol. (A) Representative photomicrographs of the SC of rats that performed the spatial discrimination task after injecting saline (left) and muscimol (right). # indicates the location of injection tip. Black lines cover the regions where c-Fos expression in glial cells was observed. Black dotted lines indicate the border of deep layers of the SC. Scale bars, 1 mm. (B) Coronal diagrams (adapted from Paxinos and Watson, 1986) showing the location of injection sites and the spread (shown by shaded areas) of muscimol within anterior (left) and posterior (right) parts of the SC after anterior-biased (n = 9) and posterior-biased (n = 4) injections, respectively. The injection sites are indicated for the saline-injected (left) and the muscimol-injected (right) hemispheres. Each shaded area indicates the area where suppression of c-Fos expression was observed in each animal. (PDF) [file pone.0025283.s003.pdf]
